# Supplementary material for: Enhanced multi-horizon photovoltaic power forecasting: A novel approach integrating ICEEMDAN decomposition with hierarchical frequency neural networks
Source: PLoS One. 2025 Nov 14;20(11):e0334828. doi: 10.1371/journal.pone.0334828 (PMC12617902; doi:10.1371/journal.pone.0334828)
Supplement: Attachment — Submitted filename: Response to Reviewers.docx [file pone.0334828.s001.docx]

College of Computer Science and Technology
Shenyang University of Chemical Technology
No. 11, Shenyang Economic and Technological Development Zone
Liaoning Province, China

Dear Dr. Sibarama Panigrahi, Academic Editor, and Esteemed Reviewers,

Thank you for your invaluable time and insightful feedback on our manuscript (**PONE-D-25-18605**). We are sincerely grateful for the constructive comments, which have been instrumental in significantly enhancing the quality, rigor, and clarity of our research. We have carefully considered every point raised by the Academic Editor and both Reviewers. Although the recommendation was for minor revisions, we have embraced this as an opportunity to substantially strengthen the manuscript. We have revised the paper with the diligence and standards of a major revision, aiming to fully address every suggestion and further enhance the scientific and innovative contributions of our work to ensure it meets the high standards of ***PLOS ONE***.

A key enhancement, prompted by Reviewer #2's insightful comments, is the complete restructuring of our experimental framework to incorporate a comprehensive uncertainty quantification analysis. We have adopted a quantile regression-based approach to generate probabilistic prediction intervals, which we believe adds significant practical value for risk-aware decision-making in real-world power systems. Consequently, the median of the probabilistic forecast now serves as our new point prediction baseline for comparison with state-of-the-art models. This methodological shift has naturally led to updated numerical results in our tables and figures, reflecting a more robust and scientifically rigorous evaluation.

Furthermore, in response to the invaluable feedback, we have undertaken a comprehensive and substantial revision of the manuscript. Our efforts were focused not only on addressing each specific comment but on elevating the overall scientific contribution, rigor, and practical relevance of our work, treating this revision with the diligence and standards of a major overhaul. The key enhancements are detailed below:

**1. Re-architected Experimental Framework: From Point Prediction to Comprehensive Uncertainty Quantification**

In direct response to the reviewer's core concern regarding practical risk assessment, we have **fundamentally expanded** our study from traditional point predictions to probabilistic forecasting. We have introduced a novel and comprehensive **uncertainty quantification framework** based on Quantile Regression and the Pinball Loss Function, with the detailed methodology is now elaborated in the new **Section 3.4**.

Under this framework, we have conducted in-depth experiments to evaluate the model's prediction interval performance **under diverse weather conditions (stable, fluctuating, and abrupt)**. The new **Section 5.5**, **Table 11**, **Figure 12**, and **Figure 13** systematically present the interval coverage (PICP) and width (PIAW), and include a detailed analysis of the non-Gaussian error distributions, greatly enhancing the practical value of our model.

As a result of this major methodological shift, we now use the **median of the probabilistic forecast as the new, more robust point prediction baseline** in all comparative experiments. This change is the root cause for the updated numerical results and figures throughout the paper and ensures our comparisons are made on a more scientific and equitable basis.

**2. Deepened Model Interpretability and Multi-Dimensional Performance Evaluation**

To "open the black box" of the model, we have added an **in-depth visual analysis of the attention mechanism**. As shown in the new **Figure 6** and discussed in **Section 5.2.1**, we intuitively demonstrate through comparative heatmaps how the proposed IRPE mechanism dynamically adjusts its focus on time steps during critical scenarios like peaks, troughs, and power ramps, thus validating its intrinsic ability to capture complex dynamics.

In response to the reviewer's considerations on practical utility, we have included a **quantitative assessment of computational complexity and scalability**. The revised **Tables 8 and 9** now contain explicit comparisons of model parameters (Params, MB) and training times for all models across different forecasting strategies, providing a transparent reference for the model's cost-effectiveness.

To further strengthen the statistical reliability of our conclusions, we have provided a more detailed presentation and discussion of the **30-round Wilcoxon signed-rank test results** (**Section 5.4**, **Table 10**, **Figure 10 & 11**). This provides powerful statistical evidence for our model's exceptional stability against the inherent randomness of the algorithms.

**3. Strengthened Scientific Rigor and Innovative Arguments**

We have fundamentally sharpened the arguments for our model's **novelty**. The new **Table 1** now systematically provides a deconstructive comparison of the ICIAL model against the latest hybrid models across multiple dimensions, including signal decomposition method, frequency-stratified modeling, and positional encoding, making its unique contributions clear.

To improve reproducibility, we have greatly expanded the **theoretical justification** for our architectural choices in the **Methods** section and have provided explicit implementation details and parameter settings for both the **ICEEMDAN algorithm** and the **PSO-based hyperparameter optimization** process (**Table 2 & 4**), addressing a key request for transparency from Reviewer #1.

Furthermore, we have added a new discussion in the final paragraphs of the **Conclusion (Section 6)** exploring the framework's **generalization potential** for other renewable energy forecasting tasks (e.g., wind) or other complex time-series domains (e.g., grid load forecasting), broadening the impact of our research.

**4. Full Commitment to Open Science and Reproducibility Principles**

In a major step towards open science and full reproducibility, and to comply with PLOS ONE's data policy and reviewer requests, we have established a **public GitHub repository**. This repository provides open access not only to all implementation scripts, model weights, and hyperparameter configurations but also, crucially, to the **"minimal dataset"** required for any researcher to fully replicate our results.

We have explicitly provided the link to this repository **(link:** [**https://github.com/Lin0v3/ICIAL-PV_Forecast**](https://github.com/Lin0v3/ICIAL-PV_Forecast)**)** in the updated **Data Availability Statement** of the manuscript, ensuring that our work can be validated, reproduced, and built upon by the scientific community.

**5. Meticulous Language and Formatting Polish**

Finally, the entire manuscript has undergone a **meticulous, line-by-line professional language edit** to enhance clarity, precision, and academic professionalism, with special attention paid to correcting the long sentences and redundancies pointed out by the reviewers. Every part of the manuscript, including all figures and tables, has been reformatted to strictly adhere to the official *PLOS ONE* templates and style guidelines, ensuring a professional final presentation.

Herein, we provide a meticulous point-by-point response to each comment raised by the Academic Editor and the esteemed Reviewers. For your convenience and to ensure full transparency, all modifications made to the original manuscript have been clearly marked in the "Revised Manuscript with Track Changes" file, using standard strikethroughs for deletions and red underlining for additions.

We are sincerely grateful for the opportunity to improve our paper based on your expert guidance. We believe that the substantial revisions we have implemented have fully addressed all concerns and significantly enhanced the scientific rigor, clarity, and overall impact of our manuscript. We hope the revised manuscript is now suitable for publication in *PLOS ONE*.

**Responses to Journal Requirements**

**1. PLOS ONE Style Requirements:**

**Comment 1:** Please ensure that your manuscript meets PLOS ONE's style requirements, including those for file naming.

**Response:** We thank the editor for this reminder. We have thoroughly reformatted the entire manuscript using the official PLOS ONE LaTeX templates for both the main body and the title/authors/affiliations page. Additionally, we have verified that all file names conform to the journal's guidelines.

**2. Code Sharing:**

**Comment 2:** Please review our guidelines...and ensure that your code is shared in a way that follows best practice and facilitates reproducibility and reuse.

**Response:** We fully agree with the importance of open science and reproducibility. We have created a public GitHub repository containing the core scripts, hyperparameter settings, and the data processing pipeline necessary to replicate our study's findings. The URL for this repository will be provided in the Data Availability Statement upon acceptance.

**3. Funding Information Mismatch:**

**Comment 3:** ...grant information you provided in the ‘Funding Information’ and ‘Financial Disclosure’ sections do not match.

**Response:** We apologize for this oversight. We have carefully corrected this discrepancy. The accurate funding information and grant numbers have been provided in the cover letter, and we request the editorial office to update the online submission form on our behalf.

**4. Funding Information in Acknowledgments:**

**Comment 4:** ...funding information should not appear in the Acknowledgments section... Please remove any funding-related text from the manuscript and let us know how you would like to update your Funding Statement.

**Response:** We have removed all funding-related text from the manuscript's Acknowledgments section as requested. The corrected and complete Funding Statement has been included in our cover letter for your convenience in updating the submission system.

**5. Data Availability Statement:**

**Comment 5:** Please confirm...whether or not your submission contains all the raw data required to replicate the results of your study.

**Response:** We confirm that all data underlying our findings are fully available. In the revised manuscript, we have updated the Data Availability Statement. The DKASC dataset is publicly available and properly cited (Reference [48]). The Solar I dataset is also derived from a public competition and is cited (Reference [49]). We have made all processed data and code required to replicate our study's results, including the values used to build graphs and tables, available through our public repository.

**6. ORCID iD for Corresponding Author:**

**Comment 6:** Please ensure that you have an ORCID iD and that it is validated in the Editorial Manager.

**Response:** We confirm that the corresponding author, Jun Wang, has a validated ORCID iD in the Editorial Manager system.

**7. Reference List:**

**Comment 7:** Please review your reference list to ensure that it is complete and correct.

**Response:** We have meticulously reviewed the entire reference list for completeness and correctness. We confirm that no retracted articles have been cited. All references are up-to-date and formatted according to the journal's guidelines.

**Responses to Reviewer #1**

We sincerely thank Reviewer #1 for the constructive and detailed feedback that has helped us strengthen our manuscript significantly.

**1. Language Editing:**

**Comment 1:** The manuscript requires substantial language editing to improve clarity, eliminate redundancy, and correct grammatical and syntactical issues.

**Response:** We sincerely thank the reviewer for this crucial feedback and fully agree that clarity and precision of the language are paramount for effective scientific communication. We have taken this comment very seriously and have conducted a comprehensive and meticulous revision of the manuscript's language. Our efforts were guided by the specific issues you highlighted and included the following key actions:

**(1) Sentence Structure and Clarity:** We have carefully reviewed and restructured many sentences throughout the manuscript that were identified as overly long or convoluted. They have now been broken down into shorter, clearer, and more direct statements to improve readability and improve the logical flow of our arguments.

**(2) Elimination of Redundancy:** We have performed a thorough search to remove repetitive phrasing and tautologies. For instance, specific examples pointed out in the review, such as the phrase "prediction value prediction," have been corrected to more concise and standard terms like "predicted value" or "prediction," depending on the context.

**(3) Correction of Grammatical and Syntactical Issues:** The manuscript has been carefully proofread to correct all grammatical errors and awkward phrasing. Examples like "as shown as fig 1 shown" have been revised to standard phrasing such as "as shown in Fig 1". Furthermore, sentences that were grammatically correct but stylistically cumbersome, such as "...effectively mitigated the randomness of experimental results, ensuring the reliability of the conclusions," have been streamlined for better impact and conciseness (e.g., "this subsequently ensured the reliability of the conclusions").

**(4) Consistency and Academic Tone:** We have thoroughly reviewed the entire document to ensure consistent use of terminology (e.g., "photovoltaic," "model," "framework") and have refined the overall tone to be more formal and academic, aligning with the standards of a high-impact journal.

These extensive language edits have been applied to the entire document, from the Abstract to the Conclusion. We are confident that these changes have significantly improved the manuscript's readability and professionalism, and we hope it now meets the high linguistic standards of *PLOS ONE*.

**2. Justification and Novelty:**

**Comment 2:** Clearly distinguish the novelty of ICIAL from similar recent hybrid models (e.g., CEEMDAN-LSTM, VMD-CNN-LSTM).

**Response:** We thank the reviewer for this vital suggestion and fully agree that clearly delineating the specific innovations of our work is essential. In response, we have undertaken a significant revision of the manuscript to precisely articulate the novelty of the ICIAL model.

Our primary effort involved substantially rewriting the **Introduction and Related Work sections (Sections 1 and 2)**. This revision provides a more critical analysis of two fundamental limitations we identified in existing advanced models: (1) adherence to a "homogenized modeling" paradigm, where a single model type is uniformly applied to all signal components, thereby neglecting their distinct physical properties; and (2) inadequate representation of temporal information due to the use of less robust absolute positional encodings. To visually reinforce this analysis and provide a systematic comparison, we have also **added a new comprehensive table (now Table 1)** to the manuscript. This table offers a head-to-head comparison of our model against a wide array of recent hybrid models across key architectural dimensions, making the unique combination of features in our model immediately apparent.

These enhancements now allow us to frame our work as a direct solution to these gaps, with our model's novelty stemming from a synergistic integration of three core contributions: (1) the use of the more advanced **ICEEMDAN algorithm** to better handle non-stationarity; (2) a **novel frequency-stratified heterogeneous network** that applies different, specialized models to high and low-frequency components, respectively; and (3) the integration of **Improved Relative Positional Encoding (IRPE)** to more accurately and robustly capture complex temporal dependencies.

We are confident that these substantial revisions now clearly position our work within the current state-of-the-art, effectively distinguishing the ICIAL model from prior research, and leave no ambiguity regarding its specific and synergistic contributions to the field.

**Comment 3:** Provide more justification for model design choices such as the selection of Conv1D-BiGRU-Attention and use of IRPE over absolute position encoding.

**Response:** We thank the reviewer for this insightful comment and have accordingly expanded our methodology section to provide much stronger and more explicit justifications for our key design choices.

In the revised manuscript, we now elaborate not just on the components, but also on the entire heterogeneous design philosophy. Specifically, for the volatile, high-frequency error sequences, we justify our choice of the **ConvBiGRU-IRPE-A** architecture in **Section 3.5.3**. We explain that this complex structure is deliberate: the **Conv1D** layer is essential for capturing local, sharp fluctuations; the stacked **BiGRU** network is designed to process comprehensive bidirectional context; and the **attention mechanism** dynamically focuses on critical transient information.

Concurrently, for the low-frequency components, which are characterized by slower changes and clearer trends, we deliberately selected a more traditional **LSTM structure** (as detailed in **Section 3.6**). This choice was intentional, as a simpler model is more efficient and highly effective at capturing long-term dependencies without the risk of overfitting that a more complex architecture might introduce on a simpler signal, a concept supported by existing literature on LSTM applications. This highlights the core principle of our heterogeneous network: applying the right

**prediction tool** for the right signal component.

Furthermore, we have provided a more detailed rationale for our selection of **Improved Relative Positional Encoding (IRPE)** over absolute positional encoding in both the **Related Work (Section 2.2)** and the **Methods (Section 3.5.2)**. We now discuss the well-documented limitations of absolute encoding, referencing literature (e.g., [9], [26]) to explain how IRPE provides a more robust representation of the dynamic temporal relationships essential for time-series forecasting. We believe these detailed additions now make our design rationale exceptionally clear and well-supported.

**3. Reproducibility:**

**Comment 4:** Clarify the implementation of ICEEMDAN (e.g., decomposition stopping criteria, noise level β) and make parameter tuning steps reproducible.

**Response:** We thank the reviewer for highlighting this important point and fully agree that reproducibility is a cornerstone of rigorous scientific research. To address this, we have significantly expanded the Methods section of our manuscript to provide a transparent and detailed account of our implementation and parameter selection processes.

First, regarding the **ICEEMDAN algorithm**, we have added Table 2, which now provides a clear, step-by-step walkthrough of the implementation. As detailed in this table and the accompanying text in Section 3.1, we have explicitly specified all crucial parameters, including the number of ensembles (**I = 50**), the initial noise standard deviation multiplier (**ϵ₀ = 0.2**), and the exact formula for adaptive noise attenuation (**ϵₖ = ϵ₀ ⋅ (0.8ᵏ)**). Additionally, we have also clearly defined the three stopping criteria used in the decomposition process, ensuring that another researcher could replicate our signal preprocessing precisely.

Additionally, to clarify the hyperparameter tuning process, we now detail our evidence-based, two-stage optimization strategy in **Section 4.4**. We explain that, to balance efficiency with effectiveness and avoid a computationally prohibitive exhaustive search, we first established an initial search space for core hyperparameters based on a systematic review of recent literature (now cited as [54-57]). Subsequently, within this empirically defined range, the Particle Swarm Optimization (PSO) algorithm was utilized for efficient automated tuning.

**Table 4** now presents the exact search ranges and the final optimal values determined by PSO for key model parameters, such as the number of filters, layers, and units. We believe these detailed additions now provide a clear and reproducible pipeline for our entire methodology.

**Comment 5:** Include a public code repository or supplementary material containing core scripts, hyperparameters, and a reproducible pipeline.

**Response:** We wholeheartedly agree with the reviewer regarding the importance of providing access to the code and data to ensure full reproducibility. In line with the principles of open science and to fully address this comment, we have established a comprehensive public GitHub repository and will include the link in the Data Availability Statement. This repository is designed to provide a complete and reproducible pipeline. It contains not only the core implementation scripts for our ICIAL model but also the specific hyperparameter configurations used in our final experiments. Crucially, in response to the journal's data policy, we have also included the "minimal dataset" required to run the code and replicate the key results presented in our manuscript.

**4. Figure Captions:**

**Comment 7:** Refine figure captions and make all figures self-contained and interpretable. Several figures (e.g., Fig 3, Fig 6, Fig 9) are referenced in the text without sufficient description.

**Response:** We thank the reviewer for this valuable feedback and agree that figures should be as self-contained and informative as possible. In response, we have performed a thorough revision of all figure captions throughout the manuscript, with special attention given to the figures you mentioned. Our goal was to ensure that each caption provides sufficient context for a reader to understand the figure's purpose and content without having to search for details in the main text. For instance: (1) the caption for **Figure 1** now walks the reader through the entire three-stage workflow of the ICIAL model, explaining the function of each numbered step from feature selection to final prediction; (2) the caption for **Figure 3** has been significantly expanded to deconstruct the complex architecture, explaining both the primary data flow through the ConvBiGRU-A layers and the dynamic, iterative process of the Improved Relative Positional Encoding (IRPE) mechanism, while also defining key variables; (3) for **Figure 6**, the caption now clarifies that the figure presents a comparative analysis of attention heatmaps across four distinct operational scenarios, enabling a direct visual assessment of the model's behavior; and (4) for **Figure 9**, the caption, combined with improved in-figure labeling, now explicitly states that the plots compare multi-step forecasting results across three different forecasting strategies (MIMO, Direct, and Recursive).

We are confident that these more descriptive and self-contained captions now make all figures, especially those highlighted by the reviewer, much clearer and more accessible.

**Responses to Reviewer #2**

We are very grateful to Reviewer #2 for the thorough review and the encouraging comments on the strengths of our work. The weaknesses identified have guided us in making substantial improvements to the manuscript.

**1. Lack of Uncertainty Quantification:**

**Comment 1:** The manuscript primarily reports point predictions. In real-world energy systems...quantifying uncertainty (e.g., prediction intervals) is essential for risk-aware decision-making.

**Response:** We are very grateful to the reviewer for this excellent and critical suggestion. We agree that quantifying uncertainty is essential for the practical application of any forecasting model in real-world energy systems. Acting on this feedback, we have undertaken a fundamental enhancement of our study by incorporating a comprehensive **uncertainty quantification framework based on quantile regression**.

To this end, we have introduced a new methodology section, **Section 3.4**, which details the principles of quantile regression, the pinball loss function used for training, and the standard evaluation metrics for probabilistic forecasting: **Prediction Interval Coverage Probability (PICP)** for assessing reliability and **Prediction Interval Average Width (PIAW)** for measuring sharpness.

A new results section, **Section 5.5**, is now dedicated to presenting and analyzing the uncertainty quantification results. This includes **Table 11**, which reports detailed PICP and PIAW values at various confidence levels, demonstrating that our model achieves excellent calibration (e.g., 94.01% PICP for a 95% confidence level at the T+24 horizon on the DKASC dataset). We have also added **Figure 12**, which visualizes these 95% confidence prediction intervals, showing that they effectively envelop the actual power values, during under abrupt weather changes. Furthermore, **Figure 13** provides an in-depth analysis of the prediction error distributions, visually confirming the non-Gaussian, heavy-tailed nature of the errors and thus validating our choice of quantile regression.

Crucially, as a result of this more advanced framework, we now use the **median (50th percentile) of the quantile regression as the new, more robust point prediction baseline** for all our multi-step comparisons. We believe this not only fully addresses the reviewer's concern but also significantly elevates the scientific rigor and practical relevance of our entire study.

**Generalization to Other Energy Domains:**

**Comment 2:** ...the manuscript could benefit from a brief discussion on whether this hybrid framework is generalizable to other renewable sources such as wind or hydroelectric power forecasting.

**Response:** We thank the reviewer for this insightful comment, which prompted us to consider the broader impact and applicability of our proposed framework. We agree that discussing generalizability is crucial. In response, we have added a new discussion on this topic in the final paragraphs of the **Conclusion (Section 6)**.

In this new section, we argue that the ICIAL model's core advantage stems not from domain-specific features but from its universal architectural principles, making it highly generalizable. We elaborate on two key points: First, the **ICEEMDAN algorithm** at the heart of our preprocessing, is a domain-agnostic signal decomposition tool, capable of effectively handling any non-stationary time series, be it from energy, finance, or climate science. Second, the principle of **hierarchical frequency modeling**—using a simpler model (like LSTM) for stable, low-frequency trends and a more complex, specialized model (like ConvBiGRU-IRPE-A) for volatile, high-frequency fluctuations—is a fundamental approach to analyzing complex systems and is not confined to photovoltaics.

Building on this rationale, we now explicitly suggest the framework's potential for transfer to other challenging prediction tasks. This includes direct applications in renewable energy, such as **wind power forecasting** (which faces similar intermittency challenges), as well as extensions to other fields requiring high-precision forecasting, including **grid load forecasting**, **financial market analysis**, and **climate modeling**. We conclude by stating that the exploration of these diverse applications is now a key direction for our future research, and we are grateful for the suggestion to broaden our perspective.

**3. Interpretability of Attention Scores:**

**Comment 3:** While the attention mechanism is well-explained, the actual interpretability of attention weights in the context of PV data is not illustrated.

**Response:** We thank the reviewer for this excellent suggestion to enhance model transparency. We agree that illustrating the interpretability of the attention mechanism provides crucial insights into the model's inner workings. To address this, we have added a **new visualization (Figure 6)** and a corresponding **in-depth analysis in Section 5.2.1** dedicated to interpreting the model's self-attention scores in the context of PV data.

This new section provides a visual, comparative analysis of the self-attention heatmaps for our ICIAL model against baseline models across four distinct and representative operational scenarios: Average, Peak, Trough, and Ramp. The analysis of these heatmaps reveals several key insights into our model's behavior. For instance, we demonstrate that the attention patterns of the ICIAL model are consistently **sharper and more focused** along key diagonals. We attribute this to the adaptive weighting enabled by the **Improved Relative Positional Encoding (IRPE)** mechanism, which effectively amplifies critical periodic dependencies while suppressing irrelevant noise.

Most importantly, the analysis highlights the model's **dynamic adaptability**. In the "Ramp" scenario, where PV power fluctuates rapidly, the ICIAL model dynamically intensifies its attention on the main diagonal, indicating a shift in focus to more recent historical data to accurately capture the slope and local trends of the change. This contrasts with the more static attention patterns of the baseline models under the same conditions. This analysis provides tangible evidence of how our model learns and adapts to different temporal dependencies, directly linking the architectural design of IRPE to the model's superior predictive performance.

**4. Computational Complexity and Scalability:**

**Comment 4:** The manuscript does not discuss the computational cost of the proposed method...a brief complexity analysis or comparison in runtime versus baselines would add practical value.

**Response:** We fully agree that a discussion of computational costs is a valuable addition for assessing the practical viability of our model. In response to this suggestion, we have integrated a new, detailed discussion of computational overhead into our multi-step forecasting analysis in **Section 5.2.3**. To support this, the results tables (**Table 8 and Table 9**) now include new columns that transparently report the "Params(MB)" and "Training Time" for our ICIAL model and all baseline models across different strategies.

The analysis presented in this text discusses the clear trade-offs between different forecasting strategies. For instance, we highlight that the Direct strategy is computationally prohibitive, and we provide a concrete example from our findings: for the T+24 forecasting task on the DKASC dataset, our ICIAL model under the Direct strategy requires **four times the parameters and nearly four times the training time** compared to its efficient MIMO counterpart. Additionally, we offer a balanced perspective on our model's intrinsic cost, noting that while it is not the lowest, its overhead (7.13 MB of parameters and ~39 minutes of training) is comparable to other advanced models like Informer.

Ultimately, this new analysis demonstrates that our proposed ICIAL model, particularly when paired with the superior MIMO strategy, achieves an effective and highly favorable balance between state-of-the-art predictive performance and acceptable computational costs, thereby reinforcing its suitability for real-world applications.

**5. Hyperparameter Selection Rationale:**

**Comment 5:** Although PSO is used, detail how initial hyperparameter ranges were chosen...to improve reproducibility.

**Response:** We thank the reviewer for highlighting this excellent point on improving reproducibility. We agree that providing a transparent rationale for hyperparameter selection is crucial. To address this, we have added a new section, **Section 4.4**, which is now dedicated to explicitly describing our two-stage hyperparameter selection strategy.

In this section, we first explain the reasoning for our approach: to balance computational efficiency with the effectiveness of the tuning process, as a fully exhaustive search would be computationally prohibitive. Our first stage, therefore, involved establishing an **evidence-based initial search space** for the core model hyperparameters. This was not an arbitrary process; as we now detail, these ranges were defined through a systematic review of recent and relevant literature on deep learning for photovoltaic forecasting, and we have added new citations **([54-57])** to support this literature-informed foundation. The exact search ranges for parameters like filters, layers, and units are now transparently presented in **Table 4**.

In the second stage, with a reasonable search space defined, we employed the **Particle Swarm Optimization (PSO) algorithm** for efficient, automated fine-tuning to pinpoint the optimal parameter combinations within these ranges. The final, optimal values discovered through this process are also reported in **Table 4**. We are confident that this detailed, two-stage methodology now provides a clear and reproducible roadmap for our hyperparameter selection process.

**6. Suggested Citations:**

**Comment 6:** Suggested Citations for Contextual Enhancement.

**Response:** We thank the reviewer for providing these suggested citations to help enhance the manuscript's context. In line with the editor's note indicating that these were not mandatory, we have carefully evaluated all five suggested articles for their relevance and potential to enrich our work.

Following this review, we found two of the suggestions to be particularly valuable for strengthening our manuscript. We have incorporated the article with DOI: 10.1016/j.eswa.2023.122147 as **Reference [15]** in our revised manuscript. This work, which discusses nature-inspired optimization algorithms and their validation methods, provides valuable context for our own use of Particle Swarm Optimization (PSO) and the subsequent statistical testing with the Wilcoxon signed-rank test, thereby reinforcing our study's methodological discussion. Additionally, we found the article with DOI: 10.32604/cmc.2022.028550 to be a helpful, contemporary example of an advanced LSTM application. We have therefore added it as **Reference [47]** in **Section 3.6.1** to support our foundational description of the LSTM architecture, which we employ for low-frequency series forecasting.

We believe that these additions, prompted by your suggestions, have enriched our manuscript's discussion and better contextualized our methods within the current literature. We are grateful for your helpful pointers.

In closing, we would like to express our deepest gratitude to the Academic Editor and both Reviewers. Your dedication, rigorous feedback, and insightful guidance have been instrumental in transforming this manuscript. The revision process has proven to be incredibly valuable, pushing us to not only address specific concerns but also to fundamentally strengthen the scientific rigor, clarity, and overall contribution of our work. We are confident that the manuscript is now a much stronger and more complete piece of research as a direct result of your input. We respectfully resubmit our work for your consideration and sincerely hope that it now meets the high standards for publication in PLOS ONE.

Sincerely,

Jun Wang Corresponding Author

College of Computer Science and Technology

Shenyang University of Chemical Technology

Email: wj_software@hotmail.com
